# Experimental Study on Liquid Metal Embrittlement of Al-Zn-Mg Aluminum Alloy (7075): From Macromechanical Property Experiment to Microscopic Characterization

**DOI:** 10.3390/ma17030628

**Published:** 2024-01-27

**Authors:** Daixin Zhang, Kaikai Cai, Jian Zheng, Haiyun Feng, Pu Song, Hongwei Hu, Zhiyuan Mao

**Affiliations:** Xi’an Modern Chemistry Research Institute, Xi’an 710065, China; 17343397424@163.com (D.Z.); ckkhhh2000@163.com (K.C.); zhengjian14@alumni.nudt.edu.cn (J.Z.); dahai99-2005@163.com (H.F.); hhw505@163.com (H.H.); 15609298362@163.com (Z.M.)

**Keywords:** liquid metal embrittlement, aluminum alloy, brittle fracture, intergranular fracture, dislocation slip

## Abstract

This study is a multiscale experimental investigation into the embrittlement of Al-Zn-Mg aluminum alloy (7075-T6) caused by liquid metal gallium. The results of the experiment demonstrate that the tensile strength of the 7075-T6 aluminum alloy significantly weakens with an increase in the embrittlement temperature and a prolonged embrittlement time, whereas it improves with an increase in the strain rate. On the basis of the analysis of the experimental data, the sensitivity of the embrittlement of 7075-T6 aluminum alloy by liquid gallium to the loading strain rate is significantly higher compared to other environmental factors. In addition, this study also includes several experiments for microscopic observation, such as Scanning Electron Microscope (SEM) observation, Energy-Dispersive Spectrometer (EDS) spectroscopy, and Electron Back Scatter Diffraction (EBSD) analysis. The experimental observations confirmed the following: (1) gallium is enriched in the intergranular space of aluminum; (2) the fracture mode of 7075-T6 aluminum alloy changes from ductile to brittle fracture; and (3) the infiltration of liquid gallium into aluminum alloys and its enrichment in the intergranular space result in the formation of new dislocation nucleation sites, in addition to the original dislocations cutting and entanglement. This reduces the material’s ability to undergo plastic deformation, intensifies stress concentration at the dislocation nucleation point, and, ultimately, leads to the evolution of dislocations into cracks.

## 1. Introduction

The phenomenon known as liquid metal embrittlement occurs when a metallic substance comes into contact with a liquid metal. This causes the mechanical properties of the metal to deteriorate; for example, a decrease in plasticity, tensile strength, ductility, and fracture strength can be observed [1]. Researchers first observed the embrittlement effect of mercury on brass in the 1920s [2]. Over the next few decades, researchers discovered instances of liquid metal embrittlement in various solid–liquid metal systems, such as Al-Ga, Ni-Bi, Cu-Bi, Cu-Hg, Fe-Pb, Fe-Zn, and others [2,3,4,5,6]. Because iron and steel are widely used in industrial production and daily life, extensive research has been conducted on the phenomenon of liquid metal embrittlement in iron [7,8,9,10].

The academic community’s understanding of the embrittlement mechanism is becoming increasingly detailed as materials science and observation equipment advance. This is due to the gradual penetration of the mechanism’s macrorepresentation into the microscopic study of liquid metal embrittlement. This integration establishes the basic embrittlement mechanism of the theoretical system from a microscopic to a macroscopic level [11,12,13]. The theoretical framework includes the creation of intergranular atomic incursions at the atomic scale and the hypothesis that liquid metal atoms can penetrate at a specific speed. This penetration lowers the Intergranular bonding energy, causing the grains to dissociate and become debonded, ultimately, leading to local failure. At the crystal scale, the impact of liquid metal on microcracks in metal materials is investigated, and a theoretical model of intergranular cracking in conjunction with microfracture mechanics is developed. It is suggested that liquid metal causes a decrease in the metal’s surface energy, which lowers the energy needed for the propagation of microcracks and causes the brittle fracture of materials under tensile stress. 

The mechanism of liquid metal embrittlement can be explained from the perspective of atomic motion in the crystal by the liquid metal’s promotion of microcrack propagation. Metal materials are always full of microscopic cracks and flaws. Stress concentration phenomena will manifest at these cracks or faults under the influence of stress. The material in the vicinity of the crack tip will fail when the local stress exceeds a particular value, causing the crack to widen and, ultimately, resulting in material fracture. The liquid metal acts at the dislocation concentration, causing the local material that could withstand a specific tensile stress to fail before the dislocation moves, the local grain boundary to crack and separate, the appearance of a new separation surface, and the subsequent creation of a new stress concentration. The material will fail once more at the stress concentration, causing the metal crystal surface to crack once more. This positive feedback will eventually cause the cracks to propagate.

When investigating the behavior of liquid metals within metallic structures through the utilization of electron microscopy, previous research has demonstrated that liquid metal atoms are capable of permeating solid metals [14]. By employing electron microscopy, Sigle et al. [12] successfully observed the infiltration of gallium atoms into aluminum intergranular regions following the injection of gallium. Research findings by Bosch et al. [15] revealed that liquid zinc in αFe has the ability to permeate steel substrates by utilizing the gaps present between grain boundaries.

Research on the embrittlement effect of gallium on aluminum has been conducted in the literature [16]. When gallium atoms enter the intergranular phase of aluminum, a much weaker aluminum–gallium metallic bond is formed, requiring less energy to break than the aluminum–aluminum metallic bond [17]. The literature suggests that in certain systems, such as bismuth–nickel and bismuth–copper, intergranular or two-dimensional grain-boundary phase complexes occur. These complexes are much weaker than the base material, which can result in premature material failure. The atomic radius, the shape of the electron layer, and the number of free electrons all influence the strength of metallic bonds. These bonds are formed through electrostatic attraction and do not possess a fixed binding energy, unlike chemical bonds. To compare the bond energies of different metallic bonds, Kelley [16] determined the relative values of these energies by comparing the electronic energies of multiple pairs of solid–liquid metals.

The brittle fracture process of metal materials has been studied and documented in the literature [17,18,19,20]. The academic community generally understands this mechanism as follows: Metal materials often contain numerous small cracks or defects. Under stress, these cracks and defects experience stress concentration, where the local stress reaches a critical value. As a result, the crack tip in the surrounding region fails, leading to crack propagation and eventual material fracture.

Liquid metal promotes the growth of microcracks in the mechanism. It acts as a dislocation-concentration agent, facilitating the movement of dislocations and enabling the material to withstand a certain level of tensile stress before experiencing localized material failure. This leads to the cracking of nearby grain boundaries and the formation of a new area of stress concentration, ultimately resulting in additional material failure. The surface of the metal crystal then cracks again, creating a positive feedback loop that, ultimately, leads to the propagation of the crack. From a macroscopic perspective, the material in the load below the yield stress is compromised when external forces, such as defects and cracks, cause the local stress to exceed a certain threshold. When the local stress exceeds the material’s yield limit, the first region to fail is often the one with the highest stress concentration, which is typically located at the crack tip [21]. This high stress near the crack tip causes the crack to start expanding, resulting in crack propagation. According to a mechanism proposed by Rostoker [22], a crack can propagate under the influence of liquid metal if there is sufficient stress. The crack originates at the intersection of the dislocation stacking and the free surface. The presence of liquid metal reduces the surface energy barrier, making it easier for the crack to propagate.

Current research on the liquid metal embrittlement of aluminum alloys is being conducted at both macroscale and microscale levels in various disciplines and perspectives to provide accurate interpretations of liquid metal embrittlement. However, there are few examples that simultaneously study the macroscopic damage and microscopic effects of liquid metal embrittlement. In addition to providing research assistance in understanding the mechanical behavior of this complex material, conducting multiscale analyses and studying similar materials can contribute to a more comprehensive understanding of the mechanisms behind liquid metal embrittlement.

In this study, liquid gallium metal and 7075-T6 aluminum alloy were utilized to experimentally observe liquid metal embrittlement. The orthogonal test method was used to design the embrittlement conditions by considering the embrittlement temperature, action time, and material strain rate as the factors to be examined. Through mechanical property testing, this study examines the embrittlement effect of liquid gallium on 7075 aluminum alloy and investigates the relevant factors. On this basis, observational experiments were carried out on its microstructure, analyzing the main dynamics and action modes, and the microstructure was characterized using a Scanning Electron Microscope (SEM), Energy-Dispersive Spectrometer (EDS), and Electron Back Scatter Diffraction (EBSD). From the standpoint of dislocation motion, an explanation and validation of the liquid metal embrittlement mechanism are provided.

## 2. Materials and Methods

The study’s experiments were divided into two sections: a macroscopic mechanical property test and a microscopic characterization observation experiment. The former utilized an electronic universal testing machine (CSS44100; CIMACH, Changchun, China) and a separate Hopkinson tensile bar (developed by Northwestern Polytechnical University’s Impact Dynamics Research Group, Xi’an, China) in accordance with variations in the strain rate under experimental conditions. The latter employed an energy spectrometer and a SEM for analysis purposes.

### 2.1. Materials Selection

Current research indicates that a number of metals, such as mercury, indium, gallium, and zinc, among others, have a clear embrittlement impact under specific circumstances. However, because of concerns about experiment viability and safety, gallium has been selected as the liquid metal for this investigation. With an atomic weight of 697.723; a silvery-white, flowable metal form at ambient temperature; and a metallic sheen, gallium has a melting point of 29.76 °C.

The most common super-hard aluminum alloy, 7075-T6, is part of the Al-Zn-Mg-Cu system of alloys. The T6 heat-treatment process consists of solution treatment (793 K-8 h)→water quenching→natural aging (12 h)→artificial aging (433 K-6 h). The 7075-T6 alloy is easy to process, has good corrosion resistance and high strength, and is used in a wide range of mechanical equipment, daily life, and aerospace applications.

Table 1 displays the physical characteristics of the aluminum alloy employed in this investigation. The internal stress of T6 will be relatively large, and it will be deformed during processing. The most suitable state for processing should be T651, which is stretched on the basis of T6 to eliminate the internal stress.

### 2.2. Sample Design

Quasi-static and dynamic tensile testing were conducted simultaneously in this investigation. The primary area of investigation was the change in tensile strength of the material’s tensile strength. Consequently, in the experiment, the material needed to be loaded until it fractured. The experimental apparatus, however, places restrictions on the dynamic experiment. The amount of tensile stress that will be produced increases with the thickness and strength of the sample. Nevertheless, there are restrictions on the split Hopkinson bar system. Therefore, separate test samples were designed for dynamic studies in order to gather high-quality experimental data.

**Figure 1:**
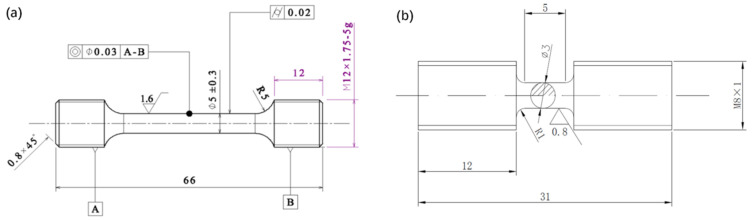
Sample design: (**a**) quasi-static stretching; (**b**) dynamic stretching.

The variation in specimen size will not impact the accuracy of the experimental results, as illustrated in Figure 1. This is because the processed data will more accurately reflect the tensile strength of the material in the center portion of the specimen.

### 2.3. Work Conditions Design

The effect of liquid metal on the target material was thoroughly examined, and the test index is the tensile strength of the target material. The impacts of three factors—embrittlement time, embrittlement temperature, and strain rate—on the test index were examined. These factors were taken to be at three different levels, as indicated in Table 2.

For the experimental conditions of three factors and three levels, a metal sample must undergo 27 operational conditions. At least 54 trials must be carried out, considering the need to repeat tests in order to reduce errors. Consequently, the experimental conditions were simplified by using the orthogonal experimental approach [23]. 

Table 3 displays the orthogonal experiment table that was created for this investigation.

Using the orthogonal test approach, all factors and levels were retained but the number of experimental working conditions decreased to nine.

### 2.4. Experiment Method

#### 2.4.1. Mechanical Performance Experiment

To control the embrittlement treatment time and temperature, the specimen must first be treated according to the orthogonal test, as in Table 3, whereby the specimen is submerged in a container filled with liquid gallium before being quickly placed inside a vacuum drying oven to maintain warmth.

The dumbbell-shaped specimens were secured onto the Hopkinson rods and universal testing machine using threads that were engraved on both ends. This exposed the section of the specimen that had undergone embrittlement treatment, while adhesive tape protected the remaining portions. The location and area of the specimen submerged in the liquid metal were controlled. Following treatment, one-dimensional stress waves were reflected to create tensile stresses, which were then generated on the testing apparatus or, in the case of high strain-rate experiments, applied using Hopkinson rods.

During the quasi-static tensile experiments, which were conducted at low strain rates, in accordance with the experimental design, the strain at break, tensile strength, and stress–strain curves of the specimens were measured using a three-dimensional digital scattering dynamic strain gauge (developed by Xi’an Jiaotong University, Xi’an, China), which is a method for measuring the surface strain and deformation of objects. This approach determines changes in the grayscale values of the speckle domain, follows the deformation process of speckle patterns on the surface of objects, and obtains strain and deformation data of the observed object’s surface [24,25].

#### 2.4.2. Microscopic Characterization Observation Experiment

This study’s microscopic observation experiments included (1) using SEM to observe the fracture morphology and microstructure; (2) using EDS to analyze the elemental components and distribution; and (3) using EBSD to characterize the crystallographic features.

Four distinct working conditions were chosen for the specimen, which was made of slices for microscopic characterization and experimental observation, for the experiments on the macromechanical properties of the experimental group species. These conditions are numbered from No. 1 to No. 4, and their design is as shown in Table 4.

Prior to being processed by mechanical polishing and electrochemical polishing, in turn, the samples that were described by EBSD and EDS were first sampled by wire cutting, in accordance with the test parameters. Using metallographic abrasive paper with mesh sizes of 1000, 2000, 3000, 5000, and 7000 on a metallographic polishing mill, in accordance with the principle of coarse to fine, mechanical polishing was performed. An OPS silicon dioxide polishing fluid was then used to further fine polish the mirror surface. Eventually, electrochemical polishing was used to eliminate any remaining stress from the sample’s surface, and an electrolytic solution was created using a 1:9 ratio of perchloric acid to alcohol. After electropolishing the sample for 20 s with a 20 V DC controlled power source, the electrolytic solution was cleaned using an ultrasonic cleaning apparatus and vacuum stored. Following sample preparation, the sample’s microcharacters were examined under a scanning electron microscope using the EBSD and EDS systems.

Using a low-magnification SEM, the general morphology of the fracture was initially studied to capture the form of the specimen’s fracture surface. Next, a high-magnification SEM was used to observe the microscopic details of the typical region. The setting conditions of the SEM were as follows: accelerating voltage—20.00 kV; specimen tilt (degrees)—70.00°; hit rate—66.51%; and speed of acquisition—22.09 Hz.

**Figure 2:**
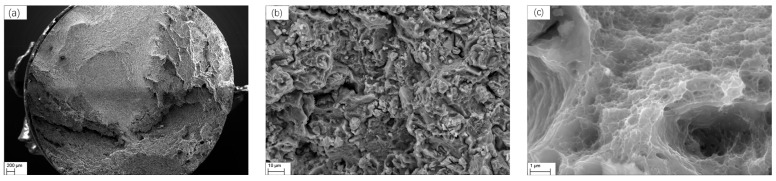
SEM images of the tensile fracture section of the 7075-T6 aluminum alloy specimen embrittled by liquid gallium (at 32 °C and 0.01 s^−1^): (**a**) magnification of 50×; (**b**) magnification of 2000×; (**c**) magnification of 25,000×.

The magnifications of the low-, medium-, and high-magnification photographs of each specimen in this investigation were determined to be 50×, 2000×, and 25,000×, respectively. An image of specimen 2# is displayed in Figure 2. To verify the significance and consistency of the results, an area of at least 96 μm^2^ was analyzed for each specimen type.

Because the experiment involved the use of a rolled alloy (7075 aluminum alloy), the rolling bar compressed the metal blank during the manufacturing process, causing the aluminum crystals to be flattened into elongated strips.

Finding a complete set of three to four adjacent grains with distinct boundaries is necessary to perform the EDS spectroscopic scans and determine the elemental content and distribution of the target. The shape, orientation, boundaries, and other details of the grains must then be analyzed using EBSD. The 2000× magnification scanning electron microscope was used for all of this work.

## 3. Results

### 3.1. Experimental Results of Mechanical Properties of 7075-T6 Aluminum Alloy

Table 5 displays the experimental tensile strength data for the 7075-T6 aluminum alloy that was embrittled by liquid gallium in this specific study.

**Figure 3:**
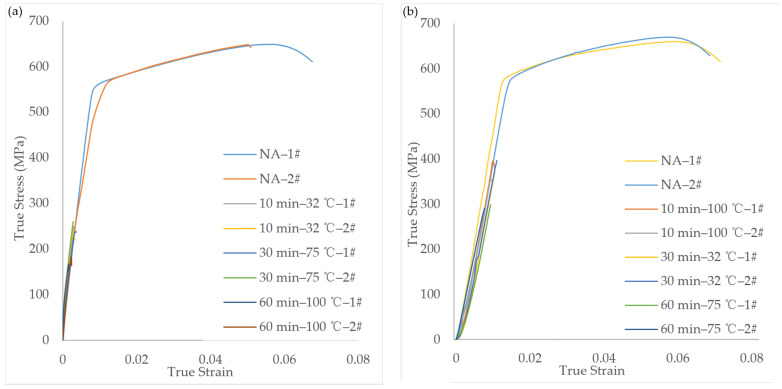
Stress–strain graphs for the quasi-static tensile tests: (**a**) 0.001 s^−1^; (**b**) 0.01 s^−1^.

The overall embrittlement effect remained consistent between the quasi-static experiments conducted at two different strain rates (0.01 s^−1^ and 0.001 s^−1^). Furthermore, the results from these quasi-static experiments do not show any significant differences. On the other hand, the reduction in the tensile strength of the dynamic test specimens is noticeably smaller than that of the quasi-static tensile specimens. Although the electronic universal testing machine and Hopkinson rod are mature and accurate instruments, the differences in the specimen shape, experimental setup, and measurement methods may still lead to inaccurate results. The solution to this problem still depends on the emergence of more widely applicable experimental equipment or innovative testing methods in the future. Taking the quasi-static experiments as an example, Figure 3 displays the stress–strain curves of specimens in the quasi-static tensile test under different operating conditions.

### 3.2. Microscopic Characterization Observation Experiment of 7075-T6 Aluminum Alloy

#### 3.2.1. 7075-T6 Aluminum Alloy Fracture Morphological Characteristics

**Figure 4:**
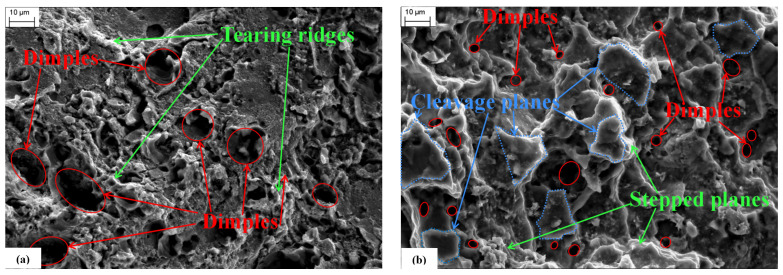
Aluminum alloy fracture morphological characteristics: (**a**) untreated; (**b**) 10 min–75 °C.

Using specimen 2# (which was not embrittled and only stretched to fracture at a strain rate of 0.01 s^−1^) and specimen 3# (which was stretched to fracture at a strain rate of 0.01 s^−1^ after being embrittled at 75 °C for 60 min), the micromorphology of the fractures under different working conditions was observed using a SEM. The results of this observation are displayed in Figure 4 at a 2000× magnification.

This fracture characteristic is typical of ductile fractures, and the fracture mode is primarily caused by the presence of dimples, mainly transgranular fractures, accompanied by a small number of intergranular fractures.

When comparing Figure 4b to Figure 4a, it is clear that the ligamentous fossa is smaller and shallower (marked in red) at a 60 min-75 °C embrittlement effect after the 7075-T6 aluminum alloy specimens experienced the same strain rate loading.

At the same time, a large number of relatively smooth cleavage planes can be seen (highlighted by blue, dashed lines in the figure). The material surrounding the dimples displays a stepped plane, and the overall morphology appears granular, which is a characteristic of intergranular fractures. These observations suggest that the material transitioned to brittle fracture at this point, primarily occurring along the crystalline fracture.

#### 3.2.2. EDS Energy Spectrum Results for 7075-T6 Aluminum Alloy

**Figure 5:**
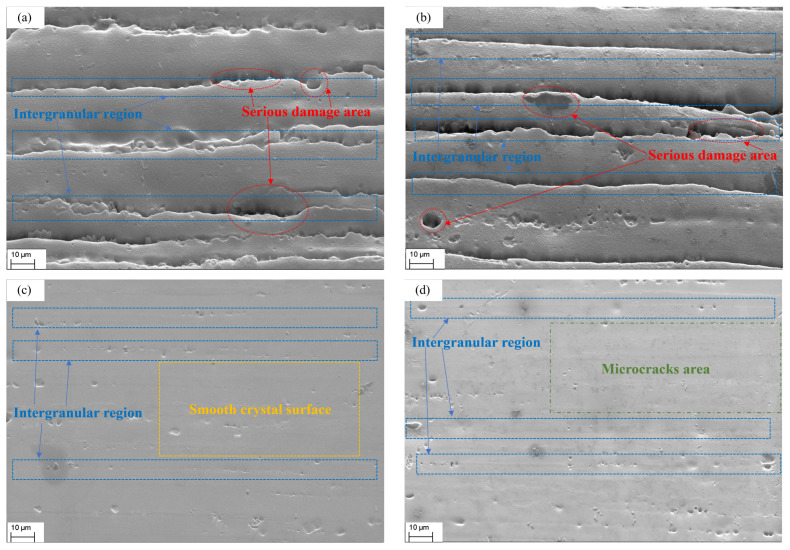
SEM images of the EDS analysis regions of the four specimens shown in Table 4: (**a**) 1#; (**b**) 2#; (**c**) 3#; (**d**) 4#.

The EDS was selected to have the same observation range as the SEM because it was used in conjunction with the SEM to examine the types and concentrations of elements in the microregion composition of the material. The SEM was performed on specimens 1# to 4# in accordance with the working condition design shown in Table 4. The purpose was to identify the more prominent three to four neighboring grains, as well as the intergranular and crystal surface areas. These areas are clearly visible in the EDS analysis under the SEM lens at a 2000× magnification, as shown in Figure 5.

The specimens embrittled by liquid gallium at 75 °C for 60 min are shown in Figure 5a,b. Their SEM images clearly display deep grooves running parallel to the direction of the crystal cell, which is the intergranular region (shown in the figure as blue, dashed boxes), which aligns with the direction of the crystal cell.

Additionally, there are black pits observed, which indicate more significant disruption to the crystal structure. This disruption is likely caused by microscopic damage or impurity enrichment, and they are distributed across both the intergranular and crystal faces (shown in the picture by the red, dashed circles). In contrast, in Figure 5c,d, in which they are not embrittled, they appear flat and smooth, with a few small pits along the intergranular region.

An energy spectrum analysis was conducted, and the mass share of each element in the region is shown in Table 6.

According to the product details provided by the supplier (Southwest Aluminum Processing Branch of China Aluminum Corporation Limited, Chongqing, China), the composition and percentage of the added elements of 7075-T6 aluminum alloy are shown in Table 7.

**Figure 6:**
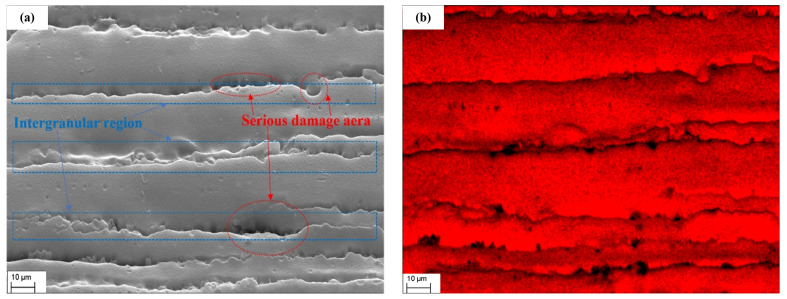

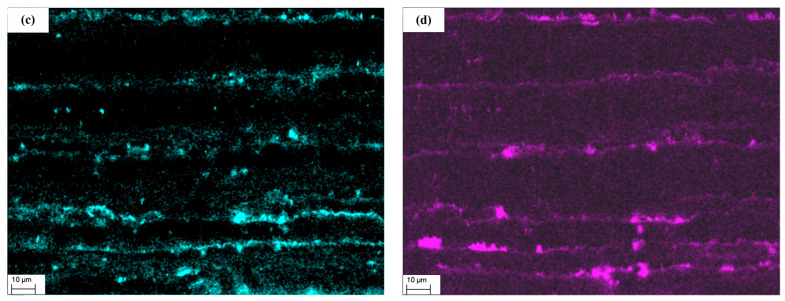
EDS spectral scans of a single element: (**a**) SEM figure; (**b**) aluminum; (**c**) gallium; (**d**) oxygen.

A significant amount of liquid gallium was detected in the EDS scanning results of the 7075-T6 aluminum alloy in this test, as shown in Figure 6 and the data in Table 6 and Table 7. 

Thus far, the observed region of this specimen has shown the presence of liquid gallium. To investigate the mechanism by which it causes liquid metal embrittlement of 7075-T6 aluminum alloy, energy spectral scans were also performed on individual elements and compared with intergranular disruption. The results of these scans are displayed in Figure 6, which compares the distributions of various individual elements with the results of the SEM scans.

Figure 6 displays the findings of an EDS scan for the elements oxygen, gallium, and aluminum. The higher the element content in the area, the more saturated the color in the graph. The black part indicates that no element of that type was discovered.

Similarly, Figure 6c shows that the distribution of the gallium element is highly consistent with its intergranular position in Figure 6a. The distribution of the oxygen element, which is abundant in clusters and mostly concentrated in the area of significant damage in Figure 6a, is depicted in Figure 6d.

Now that liquid gallium had successfully penetrated the inside of the aluminum alloy, as shown by the analysis findings of the EDS, it can be determined that the concentration of gallium element was precisely at the grain boundary by comparing the EDS and SEM images. Material embrittlement can be attributed, in part, to the enrichment of gallium elements at the grain boundary, which can be accomplished by lowering the intergranular bonding force or obstructing dislocation migration.

#### 3.2.3. EBSD Analysis Results for 7075-T6 Aluminum Alloy

Another microanalysis technique based on SEM findings is EBSD. In this work, EBSD was primarily used to investigate the role of dislocations in the liquid metal embrittlement process.

**Figure 7:**
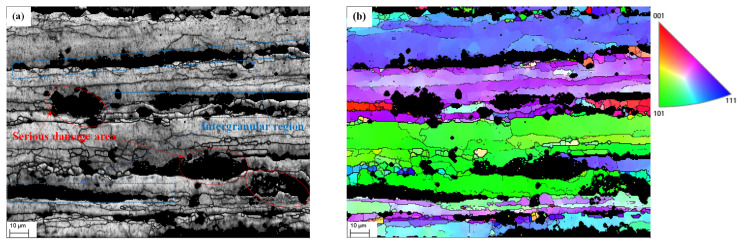
EBSD scanning results of sample 1#: (**a**) band contrast image; (**b**) inverse pole figure.

Taking sample 1# as an example, using the analysis software Channel 5 (Oxford Instruments, Oxfordshire, UK) to obtain the Band Contrast (BC) and Inverse Pole Figure (IPF), images are shown in Figure 7.

The spatial alignment in Figure 5a and Figure 7a is perfect, with the visible crystal cells essentially pointing in the same direction. The data point that the EBSD was unable to evaluate is represented by the dark portion of the picture, which is typically caused by severe and amorphous flaws. The large black area in Figure 5a can be observed at the same location in Figure 7a.

According to the results of the EBSD, the two sides of the severely damaged area were identified as different grains by the software and painted with different colors in the inverse pole figure, indicating that the position was between grains of aluminum alloy, which is consistent with the results of the SEM image analysis. The Kernel Average Misorientation (KAM) image shows that the distribution of the dislocations in aluminum alloy is different in different embrittlement and tensile stages. 

**Figure 8:**
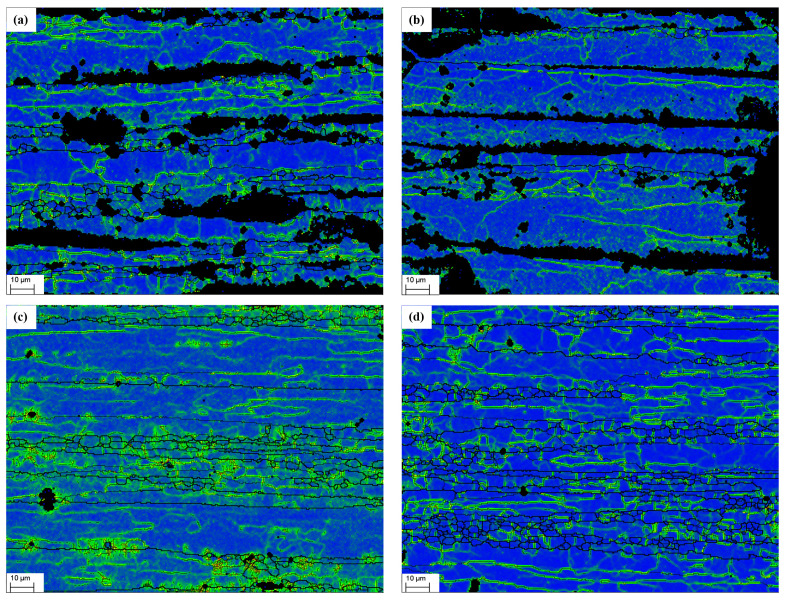
EBSD dislocation analysis images of the four specimens shown in Table 4: (**a**) 1#; (**b**) 2#; (**c**) 3#; (**d**) 4#.

The KAM results of the EBSD analysis are presented in Figure 8.

Figure 8 displays the results of the EBSD scanning of the four specimens, numbered 1 through 4. The blue portion represents the crystal surface of the aluminum alloy, whereas the black lines represent the scanned gaps, which are primarily concentrated at the intergranular position. The black part represents the area in which the EBSD could not be resolved because of local damage and impurities, whereas the green lines represent the crystalline dislocation lines.

The KAM diagram can qualitatively characterize the density and distribution of dislocations. It is a core point composed of the 24 nearest adjacent points, which is used to assign a scalar value to each point, representing its local orientation difference. The dislocation density is reflected based on the size of the local orientation difference, and areas with higher local orientation differences indicate greater plastic deformation or higher defect density. Therefore, it has a wide range of application in research such as stress corrosion cracking and grain boundary deformation coordination.

There are a large number of dislocations inside the deformed grains, which generally form the deformed grain boundaries, with an orientation difference of no more than 2° on both sides. The subgrain boundary is generally between 2 and 15°, whereas the large angle grain boundary is above 15°.

**Figure 9:**
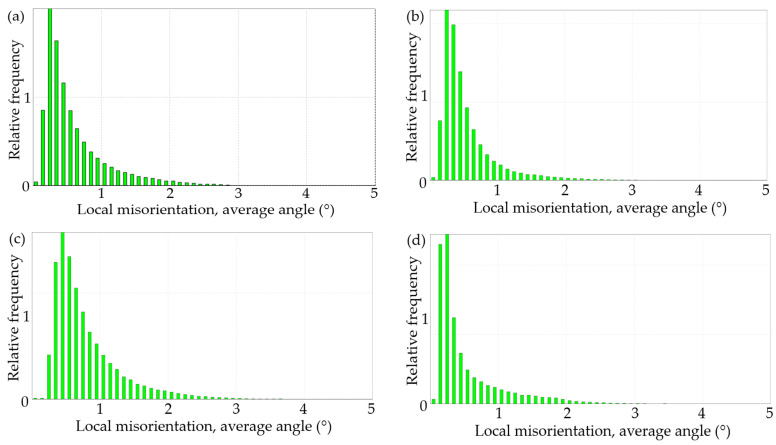
Local orientation difference distribution map for the four specimens shown in Table 4: (**a**) 1#; (**b**) 2#; (**c**) 3#; (**d**) 4#.

The EBSD can generate data-driven statistical graphs and tables while drawing KAM graphs. The local orientation difference distribution statistical graph corresponding to the KAM graph in Figure 8 is shown in Figure 9, and the statistical table is shown in Table 8.

The dislocations generally form deformed grain boundaries, and the orientation difference on both sides does not exceed 2°. Therefore, the proportion of the bar areas below 2° in Figure 9 reflects the average dislocation density in that region. Table 8 shows the statistical data generated by EBSD, which is another representation of Figure 9. The Misorientation Average Data (MAD) are the direct reflection of the dislocation density.

As can be observed in Figure 8, for the specimen that underwent no treatment, the dislocations are widely and uniformly distributed. However, when the material underwent plastic deformation due to tensile stress, the dislocations were found to concentrate in the intergranular region, with fewer dislocations observed at the crystal surface as shown in Figure 8**c**. Compared with 1# and 4#, 1# underwent liquid gallium embrittlement, whereas 4# did not. According to the data in Table 8, the introduction of liquid gallium significantly reduces the resolution ability of EBSD, with the resolution rate dropping from 97.1% to 66.5% and the effective data points dropping from 71,411 to 49,910, indicating the damage by liquid gallium infiltration to the internal structure of the crystal. 

By comparing the MAD values, it can be seen that the dislocation density of 3# is significantly higher than the other three working conditions, and while 3# and 4# did not undergo embrittlement treatment, compared 3# with 4#, the density of the dislocation under tension 4# is significantly less than for 3#, and it is mainly concentrated in the intergranular part, indicating that, at this time, the dislocation in the aluminum alloy has the ability to resist an external load through movement, and the macroscopic performance is plastic deformation.

Dislocation movement is a fundamental process in which a material undergoes plastic deformation. 

Liquid gallium embrittlement by itself does not directly alter the density and distribution of dislocations, because it is an intrinsic property of crystalline materials (only some materials cannot be detected and displayed by EBSD). The tensile plastic deformation must account for the dislocations’ contribution. This is the point at which the addition of liquid gallium turns into a material discontinuity. The introduction of liquid gallium hinders dislocation movement by forming new material interfaces, thereby reducing the plasticity of the material.

### 3.3. Analysis and Discussion of the Mechanical Properties’ Experimental Results

#### 3.3.1. Processing of Experimental Data and Overall Trend Analysis

This study adopted the orthogonal test method, which is a well-established experimental technique. Because the elements of each working condition in the orthogonal test intersect with each other, it is necessary to process the experimental data before analysis and discussion. Two types of analyses are typically available for processing data from an orthogonal test: Analysis of Variance (ANOVA) and Analysis of Range (ANOR) [26]. In this case, the more commonly used ANOR (Analysis of Range) was selected to handle the experimental data. The results are shown in Table 9.

In ANOR, *K_i_* refers to the sum of all experimental values at the *i*-th level of the factor, $K_{i}^{\prime }$ is the arithmetic mean of *K_i_*, and the *R* value of a certain factor refers to the range of all $K_{i}^{\prime }$ values of that factor, i.e., the maximum $K_{i}^{\prime }$ minus the minimum $K_{i}^{\prime }$ [26]. The *R* value describes the degree to which the factor changes affect the experimental results. The *R* value of the strain rate in this test was higher than the *R* values of the temperature and time, suggesting that the ability to influence of the three factors were as follows: B (strain rate) > A (time) > C (temperature). In other words, the greatest impact on the experimental results was caused by the change in the strain rate, followed by the temperature and embrittlement time. The embrittlement effect of liquid gallium on the 7075-T6 aluminum alloy was significantly less pronounced at low strain rates compared to the weakening of the alloy’s tensile strength at high strain rates. 

This suggests that the embrittlement effect is highly dependent on the strain rate. The influence of the strain rate on embrittlement is achieved by changing the balance between the brittle fracture rate of the materials and their own plasticity. Lowering the temperature can cause the metal to complete a transition from toughness to brittleness at a certain temperature. This is because the decrease in the temperature leads to an increase in the dislocation spacing in the martensitic structure, a significant decrease in the dislocation density, a weakening of the dislocation motion, and an increase in the energy barrier of the dislocation emission, making it difficult for the material to undergo plastic deformation through dislocation motion, thus exhibiting brittleness [27]. Low strain rates can lower the brittle–ductile transition temperature, leading to a widening of the toughness temperature range and a narrowing of the brittle temperature range, even eliminating embrittlement [28].

**Figure 10:**
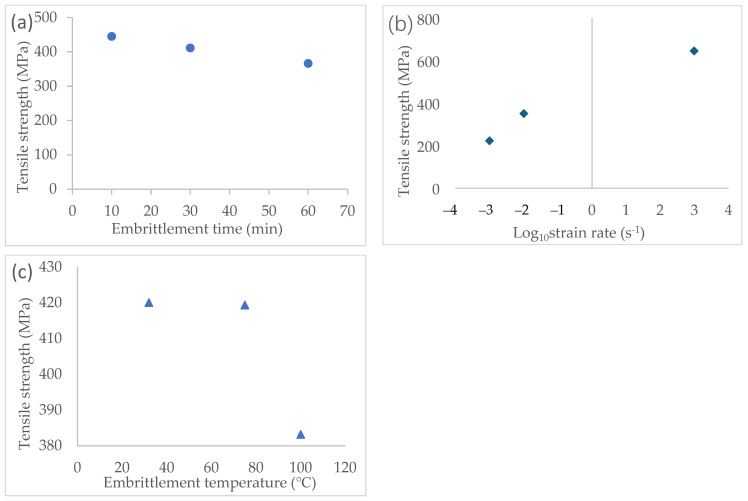
The impact of a single factor on experimental results: (**a**) embrittlement time; (**b**) ${\mathrm{Log}}_{10}strain\;rate\;(s^{-1})$; (**c**) temperature.

The analysis of range can be used to decouple the influence of a single influencing component. Figure 10 illustrates the influence of three factors on tensile strength.

The tensile strength of the 7075 aluminum alloy is significantly reduced with an increase in the embrittlement temperature and a prolonged embrittlement time, whereas the tensile strength of the specimens improves significantly with an increase in the strain rate. The three graphs in Figure 10 represent the change in the mean value of each factor; the curves represent the trend of the degree of embrittlement as a single factor increases.

#### 3.3.2. Effect of Embrittlement Temperature Variation on the Embrittlement of Liquid Metals

The mechanism by which liquid gallium penetrates the aluminum alloy is based on diffusion movement; a rise in temperature facilitates the diffusion of liquid metal atoms [29]. The higher the temperature at which liquid gallium diffuses across intergranular aluminum alloy, the more rapidly and extensively the embrittlement effect occurs [30].

In addition, metal materials will exhibit increased toughness as the temperature rises. However, this change is primarily more sensitive to high temperatures [31]. The experiment’s temperature range was 32 to 100 °C, so the increase in the metal’s toughness caused by the increased temperature was minimal within this range. In this study, the brittleness of the liquid metal increased significantly above 75 °C, which is consistent with the existing research’s conclusion.

#### 3.3.3. Effect of Embrittlement Time Variation on the Embrittlement of Liquid Metals

Because liquid metal expands the range of base metal embrittlement over time, the influence of the duration of embrittlement on the embrittlement effect tends to be more consistent. It can be assumed that the infiltration rate of liquid metal into the intergranular region will remain unchanged at a certain temperature [32], whereas the depth of infiltration and the range of embrittlement will increase over time. But it can be foreseen that the relationship will not last forever. With the degree of embrittlement, the effect of time weakens inevitably, and, ultimately, when the base metal is fully embrittled, the extension of time no longer has any role in the results of the experiments [33].

In this study, the embrittlement degree of the liquid metal showed an excellent correlation with time, and it was almost linear over a long period of time, which is also highly consistent with the conclusion in [32].

#### 3.3.4. Effect of Strain Rate Variation on the Embrittlement of Liquid Metals

It is commonly acknowledged that the tensile strength of an alloy increases with the strain rate. A material is more sensitive to the strain rate when its strength is lower. This study utilized 7075-T6 aluminum alloy, which is a type of aluminum alloy that undergoes plastic deformation immediately after reaching the yield point, resulting in strain strengthening [34]. As a result, the tensile curve of the material continues to rise even after the yield point, leading to an increase in the tensile strength due to plastic deformation. Numerous dislocations result from the plastic deformation of the metal. These dislocations are influenced by their own stress fields, causing mutual repulsion and resulting in repulsive forces. These repulsive forces contribute to the mechanical strength of the material by acting as a barrier against external loads.

The tensile strengths of the same case at strain rates of 0.001 s^−1^, 0.01 s^−1^, and 1000 s^−1^ were 648.45 MPa, 664.75 MPa, and 679.5 MPa, respectively, in the absence of any treatment. The increase in the initial tensile strength reflects the impact of the strain strengthening. By calculating the percentage of the embrittlement degree under different strain rates, it was found that the tensile strength was weakened by a maximum of 73.1% at a strain rate of 0.001 s^−1^, 43.67% at 0.01 s^−1^, and 13.89% at 1000 s^−1^.

It is evident that liquid metal embrittlement exhibits significantly weaker behavior at high strain rates compared to static stretching, both in terms of the absolute tensile strength and the relative percentage of decline observed.

### 3.4. Analysis of the Results of SEM Spectral Analysis

Figure 4b shows the micromorphology of the fracture surface of the 7075 aluminum alloy specimens after being made brittle by liquid gallium at 75 °C for 60 min. This occurred following the tensile fracture of the 7075 aluminum alloy specimens that were loaded at the same strain rate. Figure 4a shows the micromorphology of the quasi-static tensile fracture surface of the 7075 aluminum alloy specimens without undergoing liquid gallium embrittlement treatment. Large and deep tough dimples typically appear in the fracture of a material with high plasticity, whereas fine tough dimples typically appear in the fracture of a material with slightly lower plasticity [35]. The number of prominent dimples increases significantly within the same field of view.

It is consistent with the macroscopic experiments to suggest that gallium acts on the alloy to greatly impair its flexibility.

Furthermore, the shape of the tough dimple varies depending on the stress state. In tensile stress, it resembles more of a circle, whereas in shear stress it becomes elongated or even parabolic [35]. On the basis of the shape of the tough dimples on the fracture, we can determine the stress state of the microzone. The direction of the parabolic bending typically indicates the direction of the matrix that is subjected to shear stress on the side of the fracture [36]. The fact that nearly every dimple observed in this experiment was elliptical or spherical suggests that the specimens were all torn off under coaxial tension.

The white ridges around the ligamentous fossa are called tear ribs, which are a typical fracture characteristic of ductile materials. The fracture mode is mainly caused by the ligamentous fossa through crystal fracture, accompanied by a small number of fractures along the crystal [37].

Numerous smooth cleavage planes are shown in Figure 4b. These can be identified by a blue, dashed box, which indicates that the material fracture mode has now transitioned to intergranular fracture [38].

In general, the grain boundary will not crack. For an intergranular fracture to occur, there must be a trigger that weakens the bond at the grain boundary and allows the fracture to happen [39].

### 3.5. Analysis of the Results of EDS Spectral Analysis

Two primary concerns are illustrated by the results of the EDS energy spectroscopy experiment in this study:

(1) Gallium penetrated the interior of the aluminum alloy.
(2) Elemental gallium is enriched in the intergranular space of aluminum.

This indicates that liquid gallium precisely enters the interior of the 7075 aluminum alloy through the intergranular continuous penetration and invasion into the interior. As the intergranular material of aluminum alloy is replaced by gallium atoms, the intergranular bonding force of the aluminum alloy gradually decreases. This is the reason why the tensile fracture mode of the aluminum alloys shifts to an along-grain fracture, as mentioned earlier, with the embrittlement of liquid gallium.

At the same time, this also explains why the tensile fracture mode of aluminum alloys changes to intergranular fracture, as mentioned earlier, due to the embrittlement of liquid gallium.

Furthermore, as illustrated in Figure 6d, oxygen is also significantly enriched in the severely damaged and intergranular regions.

The molecular volume of various oxygen-containing gases in the air is large, and the distance between molecules is significantly larger than that of the metal atoms in the liquid metal. Therefore, it is most likely that the oxygen is the result of gallium atoms penetrating the aluminum alloy’s interior and being oxidized by the air when exposed to it following the tensile fracture [40].

### 3.6. Analysis of the Results of EBSD Spectral Analysis

The density and distribution of the dislocations within the aluminum alloy during the embrittlement process are the main focus of this study’s EBSD results.

Material undergoes dislocation slip, which contributes to its good plasticity. However, the presence of impurity elements in metal materials hinders the dislocation slip by causing dislocation movements to intersect with each other. This leads to the formation of slip steps, which result in the entanglement of dislocations and, ultimately, hinder the movement of dislocations, causing difficulties in plastic deformation and enhancing the strength of the metal.

The process of dislocation slip provides crystalline materials with additional energy to resist external stresses, as it is a fundamental mechanism of plastic deformation. When plastic deformation occurs because of external forces, the dislocation density inside the crystal increases as a result of the activation of the dislocation nucleation and propagation mechanism. With the initiation of dislocations in different slip systems and the subsequent increase in dislocation density, the mutual intersection between dislocations also increases. This leads to a significant improvement in the slip resistance and mechanical behavior. This will significantly enhance the resistance to dislocation slip, which is also well demonstrated in this experiment.

The original properties of the 7075 aluminum alloy material can be observed in specimen 4#, which did not undergo embrittlement treatment or stress loading. Its dislocation trend and crystal structure show no clear correlation, and its internal dislocations are widely and uniformly distributed.

In the necking segment of the sample observation, specimen 3# shows no embrittlement and undergoes quasi-static tensile deformation. The only difference between specimen 3# and specimen 4# is the stress loading state.

As can be seen in Figure 8c, the blue line clearly becomes thicker, as does the intergranular distribution. This indicates that during the plastic deformation caused by stretching, the dislocation density within the aluminum alloy increases, resulting in dislocation movement and a significant concentration of dislocations in the intergranular distribution.

Figure 8a,b show several regions of severe damage between the liquid metal gallium-affected crystals. The remaining blue crystal faces exhibit partial dislocation lines. The dislocation density in Figure 8d, without any treatment or stretching, is essentially the same as the dislocation distribution in Figure 8a without tensile stress. The dislocation density in Figure 8b following tensile fracture is clearly lower than in Figure 8a,d. The green lines in Figure 8b are associated with the black failure area and tend to congregate in its direction.

According to the aforementioned EBSD features, dislocations in the aluminum alloy undergo slip motion during the untreated embrittlement tensile fracture process, leading to plastic deformation of the material. Gallium introduction, however, results in a significant area of severely damaged microstructure near the grain boundaries. This severely impedes dislocation movement, and a large number of dislocations continue to aggregate and eventually develop into cracks, in contrast to the case in which the material is not embrittled. As seen in Figure 8b, dislocations traveling in various directions are dispersed by initiating fractures, resulting in a significant reduction in dislocations.

## 4. Discussion

This study focuses on the main influencing factors and sensitivity of gallium embrittlement in liquid metal of 7075-T6 aluminum alloy and attempts to explain the reasons using microscopic experiments. 

There have been many studies on the influencing factors and action laws of liquid metal embrittlement in the academic community. The research on different solid–liquid metal pairs shows that the liquid metal embrittlement effects of solid–liquid metal systems that seem to have similar chemical properties are greatly different, showing significant chemical specificity, so the selection of materials has a significant impact on the liquid metal embrittlement effect [41,42].

A given solid–liquid metal pair only shows liquid metal embrittlement sensitivity in a certain temperature range, and the occurrence of liquid metal embrittlement will be inhibited above and below that temperature range. For the system to be insensitive to temperature changes, the reasons may be that the plastic effect of a temperature rise on the metal material offsets its effect on the brittle fracture caused by liquid metal, which hinders the occurrence of brittle fracture.

Generally speaking, with the extension of the exposure time, the brittleness of the material became more significant. But not all systems are sensitive to exposure time. In some experiments, liquid metal penetrates and embrittles the target metal material very quickly; then, the whole system tends toward a stable state, and the whole embrittlement process can be completed in a few minutes [43].

The embrittlement of liquid metal can be carried out more fully by prolonging the exposure time, especially for systems with low solubility, which need longer times to form and expand cracks, but the action time is not a factor affecting the final effect of embrittlement of liquid metal, and the damage effect cannot be deepened by prolonging the time. The dependence of the embrittlement effect on temperature is only valid within a certain time range [44].

At present, most researchers focus on one kind of influencing factor on the liquid metal embrittlement. However, this research attempted to simultaneously study the three main influencing factors, while also paying attention to the relationships among different factors while studying their patterns of action. This study provides the degree of influence of the temperature, time, and strain rate on the embrittlement of liquid metals through the relationship of the R values.

## 5. Conclusions

This study investigated the changes in material characteristics, the fundamental principles and variables influencing embrittlement, and the microprocesses that contribute to embrittlement through experimental investigations on the liquid gallium embrittlement of 7075-T6 aluminum alloy at various scales. The conclusions obtained are as follows:

1. The complex physical phenomenon known as liquid metal embrittlement is influenced by several factors. Three of these factors were studied in this research: loading strain rate, embrittlement temperature, and duration of embrittlement. Nonetheless, the experiment proved that these factors have noteworthy influences on embrittlement. With regard to the combination of liquid gallium and aluminum alloy 7075-T6 chosen for this investigation, the most notable factor was the influence of the strain rate, which significantly outweighed the other two variables. The liquid metal reduced the material’s tensile strength by 73.1% at a strain rate of 0.001 s^−1^. The decrease in the strain rate at 0.01 s^−1^ was 43.67%, but at a strain rate of 1000 s^−1^, it was only 13.89%. When compared to the same material treated for embrittlement under low strain rates, the decrease in the tensile strength at high strain rates was considerably smaller, both in absolute terms and as a percentage. Therefore, it is necessary to consider preventing liquid metal embrittlement in structural components made of 7075-T6 aluminum alloy during their use. In mechanical design, components made of 7075-T6 are considered to meet the design requirements if they satisfy the criteria for strength under static stress conditions and resistance to liquid metal embrittlement.
2. The main area of aluminum alloy in which liquid gallium permeates and diffuses is the intergranular region of aluminum. The addition of liquid gallium to the intergranular aluminum alloy decreases the intergranular binding energy, leading to the formation and expansion of cracks within the intergranular space. This changes the material fracture mode, which shifts from ductile fracture, characterized by dimples, to brittle fracture along the intergranular boundaries of the aluminum alloy.
3. The infiltration of liquid gallium into aluminum alloys and the enrichment between intergranular areas also create material discontinuities, which impede the movement of dislocations. This process results in the formation of new sites for dislocation nucleation, in addition to the original processes of dislocation cutting and entanglement. It decreases the material’s capacity for plastic deformation, amplifies stress concentration at the site where dislocations form, and, ultimately, causes the dislocations to transform into cracks. These cracks release local stress by propagating, ultimately resulting in brittle fracture.

## Figures and Tables

**Table 1:** Performance parameters of 7075-T6 aluminum alloy.

Material Property	Contents
Aluminum alloy grade	7075-T651
Melting point (°C)	477~638
Density (gcm3)	2.85
Tensile strength (MPa)	591.13
Fracture strain (mm)	0.108

**Table 2:** Setting of experimental conditions.

Factor	Embrittlement Time/min	Strain Rate/s^−1^	Temperature/°C
Factor 1	10	0.001	75
Factor 2	30	0.01	100
Factor 3	60	1000	32

**Table 3:** Orthogonal experiment conditions design.

Test Number	A (Embrittlement Time/min)	B (Strain Rate/s^−1^)	C (Temperature/°C)
1	1 (10)	1 (0.001)	3 (32)
2	2 (30)	1 (0.001)	1 (75)
3	3 (60)	1 (0.001)	2 (100)
4	1 (10)	2 (0.01)	2 (100)
5	2 (30)	2 (0.01)	3 (32)
6	3 (60)	2 (0.01)	1 (75)
7	1 (10)	3 (1000)	1 (75)
8	2 (30)	3 (1000)	2 (100)
9	3 (60)	3 (1000)	3 (32)

**Table 4:** Design of the microscopic observation experimental conditions.

Sample Number	Embrittled by Liquid Gallium (70 °C, 60 min)	Stretch to Fracture (0.001 s^−1^)
1#	Yes	No
2#	Yes	Yes
3#	No	Yes
4#	No	No

**Table 5:** Experimental results of the mechanical properties of liquid-gallium-embrittled 7075-T6 aluminum alloy.

Stress Loading State	Embrittlement Condition	Sample Number	Tensile Strength (MPa)	Arithmetic Mean (MPa)	Relative Value (%)
Quasi-static loading (0.001 s^−1^)	Untreated	1	649.0	648.45	100
		2	647.9		
	10 min–32 °C	1	248.8	246.4	38
		2	244.0		
	30 min–75 °C	1	237.7	249.35	38.5
		2	261.0		
	60 min–100 °C	1	166.5	174.5	26.9
		2	182.5		
Quasi-static loading (0.01 s^−1^)	Untreated	1	659.8	664.75	100
		2	669.7		
	10 min–100 °C	1	395.7	374.45	56.3
		2	353.2		
	30 min–32 °C	1	372.0	384.15	57.8
		2	396.3		
	60 min-75 °C	1	298.3	294.7	44.3
		2	291.1		
Dynamic loading (1000 s^−1^)	Untreated	1	687.4	697.5	100
		2	707.6		
	10 min–75 °C	1	736.3	714.1	102
		2	691.9		
	30 min–100 °C	1	682.0	600.6	86.1
		2	519.2		
	60 min–32 °C	1	618.8	629.7	90.2
		2	640.6		

**Table 6:** Mass ratio of each element as obtained from the EDS energy spectrum analysis.

Element	wt%	Element	wt%
Al	81.49	Mg	1.97
C	1.81	O	1.05
Ca	0.11	Ti	0.04
Cr	0.24	Fe	0.15
Mn	0.12	Cu	2.27
Zn	5.20	Ga	5.54
Total	100.00		

**Table 7:** Composition and percentage of added elements of 7075-T6 aluminum alloy.

Element	Fe	Si	Mn	Cr	Cu	Ti	Zn	Mg	Ni	Al
Wt%	0.305	0.079	0.242	0.195	1.518	0.025	5.38	2.38	0.01	others

**Table 8:** EBSD’s data statistics on the KAM graphs for the four specimens shown in Table 4.

Specimen	Resolution Rate (%)	Effective Points	Mean BC	Min BC	Max BC	Mean MAD	Min MAD	Max MAD
1#	66.51	49910	143.68	30.00	255.00	0.49	0.05	1.99
2#	62.28	76233	145.90	32.00	255.00	0.53	0.07	2.00
3#	96.58	73391	166.79	37.00	235.00	0.67	0.15	2.00
4#	97.07	71411	192.89	38.00	252.00	0.42	0.08	1.99

**Table 9:** The results of the Analysis of Range (ANOR) of this experiment.

Test Number	A (Embrittlement Time/min)	B (Strain Rate/s^−1^)	C (Temperature/°C)	Tensile Strength/MPa
1	1 (10)	1 (0.001)	3 (32)	246.4
2	2 (30)	1 (0.001)	1 (75)	249.35
3	3 (60)	1 (0.001)	2 (100)	174.5
4	1 (10)	2 (0.01)	2 (100)	374.45
5	2 (30)	2 (0.01)	3 (32)	384.15
6	3 (60)	2 (0.01)	1 (75)	294.7
7	1 (10)	3 (1000)	1 (75)	714.1
8	2 (30)	3 (1000)	2 (100)	600.6
9	3 (60)	3 (1000)	3 (32)	629.7
$K_{1}$	1334.95	670.25	1258.15	
$K_{2}$	1234.1	1053.3	1149.55	
$K_{3}$	1098.9	1944.4	1260.25	
$K_{1}^{\prime }$	444.98	223.42	419.38	
$K_{2}^{\prime }$	411.37	351.1	383.18	
$K_{3}^{\prime }$	366.3	648.13	420.08	
R	78.68	424.72	36.9	
Factor priority	B (Strain rate) > A (Embrittlement time) > C (Temperature)			
Preferred level	A1	B3	C3	

## Data Availability

The data presented in this study are available upon request from the corresponding author.
